# Soil properties, seasonality and crop growth stage exert a stronger effect on rhizosphere prokaryotes than the fungal biocontrol agent *Fusarium oxysporum* f.sp. *strigae*

**DOI:** 10.1016/j.apsoil.2016.03.021

**Published:** 2016-09

**Authors:** Mary K. Musyoki, Georg Cadisch, Judith Zimmermann, Henry Wainwright, Fen Beed, Frank Rasche

**Affiliations:** a Institute of Agricultural Sciences in the Tropics (Hans-Ruthenberg-Institute), University of Hohenheim, Stuttgart, Germany; b The Real IPM Company, P.O. Box 4001-01002, Madaraka, Thika, Kenya; c AVRDC—The World Vegetable Center, East and Southeast Asia, P.O. Box 1010 (Kasetsart), Bangkok 10903, Thailand

**Keywords:** Maize rhizosphere, *Fusarium oxysprorum* f.sp*. strigae* inoculation, Bacterial and archaeal *amoA* gene abundance and community composition, Rhizosphere competence

## Abstract

*Fusarium oxysporum* f.sp. *strigae* (Fos) is an effective biocontrol agent (BCA) against the parasitic weed *Striga hermonthica*. It acts in the rhizosphere of several tropical cereals, where it may interfere with indigenous microbial populations. To test this impact, we assessed in a 2-season field experiment at two contrasting tropical agro-ecological sites the response of nitrifying and total indigenous prokaryotic communities in the rhizosphere of maize to the exposure of the Fos-BCA “Foxy-2”. At early leaf development (EC30), flowering (EC60) and senescence (EC90) stage of maize, rhizosphere samples were obtained and subjected to community analysis of bacterial and archaeal *amoA* (ammonia monooxigenase) (AOB, AOA) and 16S rRNA genes. Abundance and community composition of all studied genes were predominantly influenced by soil type, crop growth stage and seasonality. No major effect of “Foxy-2” was found. Notably, total archaeal community relative to bacteria dominated in the clayey soil which was linked to its strong soil organic carbon (SOC) background. Compared to bacterial nitrifiers, domination of nitrifying archaea increased towards senescence stage which was explained by biochemical differences in organic resource availability between the crop growth stages. During the short rain season, the higher archaeal abundance was mainly driven by increased availability of organic substrates, i.e., extractable organic carbon. Our findings suggested that archaea had greater rhizosphere competence than “Foxy-2” in soils with higher clay and SOC contents. We verified that “Foxy-2” in maize rhizospheres is compatible with nitrifying prokaryotes under the given environments, in particular in clayey soils dominated by archaea.

## Highlights

• Natural factors had major effects on community dynamics of microbial target groups.
• Conversely, “Foxy-2” exposed no major effect on rhizosphere microbial communities.
• Archaeal community had greater rhizosphere competence than “Foxy-2” in clayey soil.
• Compatibility of indigenous soil nitrifying prokaryotes with “Foxy-2” was verified.

## 1 Introduction

The fungal strain *Fusarium oxysporum* f.sp *strigae* (“Foxy-2”) has been acknowledged as a potent biological control agent (BCA) against *Striga hermonthica* which is parasitic to several cereals cultivated in Sub-Saharan Africa (Schaub et al., 2006, Venne et al., 2009, Elzein et al., 2010). “Foxy-2” proliferates in the crop rhizosphere and has been mainly studied regarding its virulence and mode of action (Schaub et al., 2006, Ndambi et al., 2011, Avedi et al., 2014).

The rhizosphere is a hot spot of microbial activities interacting with plants (Grayston et al., 1998), but no understanding is currently available regarding the interactions of “Foxy-2” with indigenous microorganisms colonizing the roots of cereals and environmental factors including site-specific soil and climatic conditions. This is of particular relevance since “Foxy-2” is often delivered via seed coating and proliferates subsequently along the roots where it directly interacts with other rhizosphere organisms. Thus, prior to broad-scale application of the BCA “Foxy-2” in the field, its compatibility with non-target rhizosphere microorganisms needs to be thoroughly assessed under contrasting environmental conditions (Miedaner et al., 2001, Edel-Hermann et al., 2009, Martin-Laurent et al., 2013). In the rhizosphere, “Foxy-2” must co-exist with indigenous microbial populations as well as maintain its efficacy under a range of environmental factors including seasonal alternations considering rainfall and temperature patterns, varying soil types and also crop growth stages (Girvan et al., 2003, Beed et al., 2007, Bell et al., 2009, Raaijmakers et al., 2009, Watson, 2013).

Abundance and composition of rhizosphere microbial communities are mainly shaped by rhizodeposition which is the transfer of plant-derived carbon (C) and nitrogen (N) compounds below ground (Høgh-Jensen and Schjoerring, 2001, Kandeler et al., 2002, Rasche et al., 2006a, Wichern et al., 2008, Jones et al., 2009, Fustec et al., 2011). Rhizodeposition is influenced by external factors such as soil type (properties), plant species and their growth stages, as well as climatic conditions (Rasche et al., 2006a, Hai et al., 2009, Hayden et al., 2010). Climatic conditions are of particular importance as rainfall and temperature control crop physiology, photosynthesis activity and consequently rhizodeposition shaping the root-associated microbial community (Waring and Running, 1998, Bell et al., 2009, Rasche et al., 2011).

It remains speculative, if an inoculation and proliferation of “Foxy-2” in the crop rhizosphere results in competition with indigenous rhizosphere for root exudates as critical energy sources and if such interactions are influenced by environmental factors. This assumption is corroborated by earlier studies showing bacterial populations affected by fungi leading to a distinct selection of competitive community members in the rhizosphere (Marschner et al., 2001, Strange, 2005, Cavagnaro et al., 2006). Musyoki et al. (2015) showed that “Foxy-2” inoculated into soils was compatible with nitrifying prokaryotes.

Many BCAs exhibit beneficial effects under laboratory set-ups, while such effects become inconsistent once they are evaluated under greenhouse or even field conditions (Lugtenberg and Kamilova, 2009, Avedi et al., 2014). Advanced understanding of the influence of “Foxy-2” on non-target, functionally relevant rhizosphere prokaryotes under contrasting field conditions is therefore not only essential to validate previous studies under controlled conditions (Musyoki et al., 2015). It is also important to evaluate the extent of a “Foxy-2” impact against acknowledged factors (e.g., seasonality, soil type, crop growth stage) that determine the dynamics of rhizosphere communities (Doohan et al., 2003, Rasche et al., 2006a, Rasche et al., 2011).

Bacteria and archaea are ubiquitous in soils and responsible for the decomposition and mineralization (i.e., N cycle) of organic matter (Widmer et al., 2006, Raybould and Vlachos, 2011). A critical component of the microbial driven N cycle is the nitrification step which is catalyzed by key enzymes such as the *amoA* gene encoding the α-subunit of ammonia monooxygenase (Nicol et al., 2008, Zhang et al., 2013). It has been extensively reported that abundance and community composition of ammonia-oxidizing prokaryotes (i.e., bacteria (AOB), archaea (AOA)) respond sensitively to environmental change including the exposure to fungi (Rasche et al., 2011, Raybould and Vlachos, 2011, Wessén and Hallin, 2011, Martin-Laurent et al., 2013, Pereira e Silva et al., 2013). Although these studies acknowledge the use of AOB and AOA abundance as bioindicators for soil ecosystem disturbance surveys, the effects of BCAs (e.g., “Foxy-2”) on dynamics of these functionally relevant groups in soils are yet to be understood.

The objective of this study was therefore to assess the response of ammonia-oxidizing prokaryotes to “Foxy-2” exposure and to assay these presumed effects at different growth stages of maize cultivated in contrasting environments during two cropping seasons. In addition, we have evaluated these effects against those caused by an N-rich organic input which was supposed to compensate any resource competition between “Foxy-2” and non-target rhizosphere microbial communities (Ayongwa et al., 2011, Musyoki et al., 2015). The major hypothesis was that under field conditions, natural factors such as crop growth stage, soil type (properties) and climatic conditions (i.e., rainfall and temperature patterns) expose a greater influence on the abundance and community composition of resident prokaryotic populations than the BCA “Foxy-2” in a maize rhizosphere (Buée et al., 2009, Nihorimbere et al., 2011).

## 2 Material and methods

### 2.1 Fungal biocontrol agent

The fungal strain *Fusarium oxysporum* f.sp. *strigae* (“Foxy-2”) used in this study was isolated from diseased *S. hermonthica* collected in North Ghana (Abbasher et al., 1995). Identification of the isolate was done by the Julius-Kühn-Institute, Berlin, Germany (accession number: BBA-67547-Ghana). Since then, the isolate is being preserved at −80 °C at the Institute of Plant Production and Agroecology in the Tropics and Subtropics, University of Hohenheim, Stuttgart, Germany.

### 2.2 Study site description

**Fig. 1:**
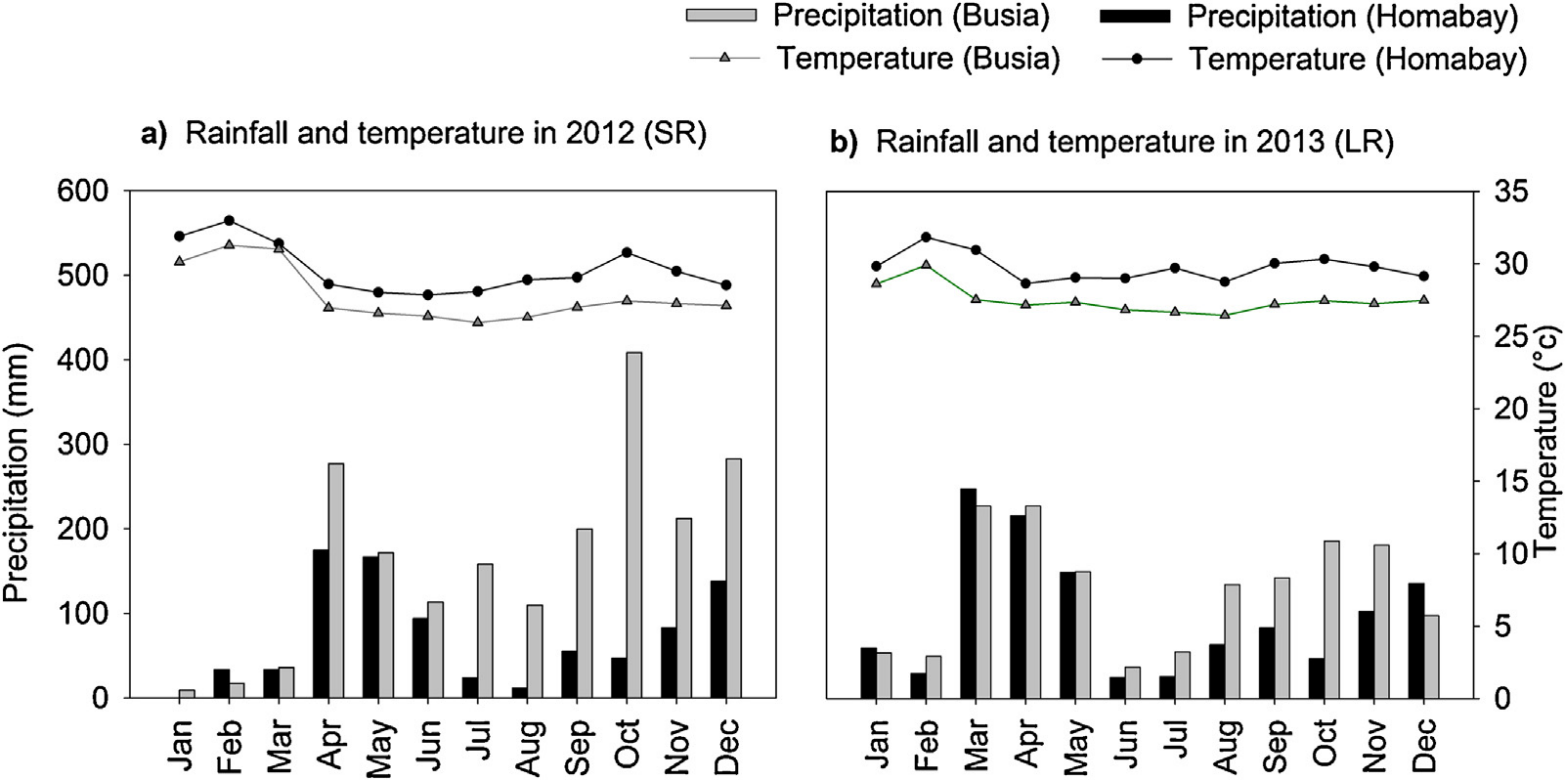
Monthly rainfall and temperature distribution during the short rain (SR) season (September 2012–January 2013) and the long rain (LR) season (March–August 2013).

The field experiments were carried out in post-entry quarantine facilities (PEQ) at Agricultural Training Centre field stations in Western Kenya (Avedi et al., 2014). Two study sites (Busia, 0°26′S–34°15′E; 1200 m above sea level (a.s.l.); Homabay, 0°40′–0°S and 0°34°50′E; 1305 m a.s.l.) were chosen because of the reported high *S. hermonthica* infestation in these areas (De Groote et al., 2005). The sites were fallow for a year before the experiment was established. The fallow in Busia consisted of short grasses (e.g., *Digitaria scalarum*), while the fallow at Homabay consisted of grasses (*Digitaria scalarum*), and weeds such as black nightshade (*Solanum nigrum*) and thorn apples (*Datura stramonium*). The study areas have bimodal rainfall patterns with two growing seasons, the first rainy season with long rains (LR) from April to August and the second rainy season with short rains (SR) from September to January. Busia received 121 and 231 mm precipitation per month during the LR and SR season, respectively, and had a mean temperature of 27 °C during both seasons (Fig. 1). Homabay received 216 and 77 mm of rainfall per month during the LR and SR season, respectively, while the mean annual temperature was 29 °C in both seasons (Fig. 1). Initial soil characterization revealed that the soil at Homabay has a clayey texture (49% clay, 19% silt, 32% sand) and contained 0.22 and 2.87% total nitrogen and carbon, respectively, while Busia soil has a clay loam texture (33% clay, 22% silt, 45% sand) and contained 0.19 and 1.57% total nitrogen and carbon, respectively.

### 2.3 Field experiment and rhizosphere sampling

The study covered two seasons (first season: SR; September 2012 to January 2013; second season: LR; April 2013 to August 2013). Maize variety WH507, commonly preferred by farmers in the study area due to its less susceptibility to *S*. *hermonthica* and also recommended for an integrated *S*. *hermonthica* control, was planted in 3 × 2.7 m^2^ plots with a spacing of 30 by 70 cm (Avedi et al., 2014). The experiment was laid out in a randomized complete block design (RCBD) with three replicates and comprised of three treatments: (i) uncoated maize and *S. hermonthica* (C, control), (ii) coated maize with “Foxy-2” and *S. hermonthica* (F + S), and (iii) coated maize with “Foxy-2”, *S. hermonthica* and *Tithonia diversofolia* residues as additional N source (F + S + T). Maize seeds were coated with “Foxy-2” (1.15 × 10^5^ colony forming units per seed) as described by Musyoki et al. (2015).

Land was prepared by hand digging and two maize seeds per hill were planted at a depth of approximately 3 cm. One Table spoonful of a *S. hermonthica* seed-sand mixture (1:4 ratio with approximately 1000 *S. hermonthica* seeds) was placed in every planting hole (Avedi et al., 2014). At sowing, all plots received a blanket application of 60 kg P_2_O_5_ ha^−1^ season^−1^ as diammonium phosphate (DAP) (NH_4_)_2_HPO_4_) to avoid any phosphorus limitation. In addition, mineral N fertilizer was split applied to treatments C + S and F + S as calcium ammonium nitrate (CaNH_4_NO_3_) at a rate of 120 kg N ha^−1^ growing season^−1^ with 1/3 and 2/3 added 3 and 8 weeks after sowing, respectively (Chivenge et al., 2011, Muema et al., 2015). For treatment F + S + T, N was applied as fresh *T. diversifolia* leaf and stem material (5 t dry weight ha^−1^ to supply similar levels to 120 kg of inorganic N) which was hand-incorporated to a soil depth of 0–15 cm at the onset of each rainy season. Two weeks after germination, seedlings were thinned to 1 per hole. Hand weeding was done after every 2 weeks for all weeds except *S. hermonthica*.

Rhizosphere samples (approximately 50 g) were collected according to standard procedures (Milling et al., 2004) at EC30 (early leaf development stage), EC60 (flowering stage), and EC90 (senescence stage) by shaking the roots of three plants per plot to remove non-rhizosphere soil. Rhizophere soil samples were scraped off the roots of sampled plants. The rhizosphere soil samples of the three representative plants per plot were then mixed to form one composite sample. Soils were freeze-dried and stored in a dark and dry place until further analysis.

### 2.4 Microbial abundance

Four hundred milligrams of freeze-dried rhizosphere soil was used for DNA extraction from each of the three replicates per treatment. Soil DNA was extracted using the FastDNA^®^ Spin for Soil Kit (MP Biomedicals, Solon, Ohio, USA) following the manufacturer’s instructions. Quality of extracted DNA was checked using 1.5% (w/v) agarose gel. DNA extracts were quantified (Nanodrop ND-1000, NanoDrop Technologies, Wilmington, USA) and stored at −20 °C for further analysis.

Abundance of total and ammonia oxidizing prokaryotic communities was estimated by DNA-based quantitative PCR (qPCR) using bacterial and archaeal 16S rRNA genes (total community) as well as bacterial and archaeal genes encoding ammonia-monooxygenase (*amoA* genes) as molecular markers (Table 1). For standard preparation, amplicons from each target gene were purified (Invisorb Fragment CleanUp kit, Stratec Molecular GmbH, Berlin, Germany), ligated into the StrataClone™ PCR cloning vector pSC-A (Stratagene, La Jolla, CA, USA) and ligation products were transformed with StrataClone Solopack competent cells (Startagene) (Rasche et al., 2011). The qPCR assays were carried out in a 25 μl reaction containing 12.5 μl of Power SYBR^®^ green master mix (Applied Biosystems, Foster City, CA, USA), 1 μl primer (each 0.4 μM), 0.25 μl T4 gene 32 protein (500 ng μl^−1^, MP Biomedicals) and 10 ng template DNA. The qPCRs were performed on a StepOnePlus™ Real-Time PCR System (Applied Biosystems) and were started with 10 min at 95 °C, followed by amplification cycles specific for each target gene (Table 1). Melting curve analysis of amplicons was conducted to confirm that fluorescence signals originated from specific amplicons and not from primer-dimers or other artifacts. Each DNA sample was processed in triplicate reactions, whereas the standard curves were generated using duplicate serial dilutions of isolated plasmid DNA containing the genes studied (Rasche et al., 2011). Gene copy numbers and reaction efficiencies (total bacteria 94% ± 8, total archaea 97% ± 3, AOB 96% ± 7, AOA 96% ± 9) were calculated using the Stepone software version 2.2.2 (Applied Biosystems) and presented per gram of dry soil.

**Table 1:** Description of primer sets, PCR ingredients and amplification details used for quantitative PCR and T-RFLP analysis.

Target group	Primer (reference)	qPCR	T-RFLP
All bacteria (16 S rRNA)	Eub338 (Lane, 1991)	95 °C 5 min	
	Eub518 (Muyzer et al., 1993)	40 cycles: 95 °C 30 s, 55 °C 35 s, 72 °C 45 s	
	8f (Weisburg et al., 1991)		95 °C 5 min
	1520r (Edwards et al., 1989)		40 cycles: 95 °C 1 min, 58 °C 30 s, 72 °C 1 min; 72 °C 10 min

All archaea (16 S rRNA)	Ar109f (Lueders and Friedrich, 2000)	95 °C 5 min	95 °C 5 min
	Ar912r (Lueders and Friedrich, 2000)	40 cycles: 95 °C 30 s, 52 °C 35 s, 72 °C 45 s, 78 °C 20 s	35 cycles: 95 °C 1 min, 52 °C 30 s, 72 °C 1 min, 72 °C 10 min

Ammonia oxidizing bacteria (AOB)	AmoA-1f (Rotthauwe et al., 1997)	95 °C 5 min	95 °C 5 min
	AmoA-2r (Rotthauwe et al., 1997)	45 cycles: 95 °C 30 s, 57 °C 45 s, 72 °C 45 s, 78 °C 20 s	40 cycles: 94 °C 30 s, 53 °C 30 s, 72 °C 1 min, 72 °C 10 min

Ammonia oxidizing archaea (AOA)	Arch-amoAf (Francis et al., 2005)	95 °C 5 min	94 °C 5 min
	Arch-amoAr (Francis et al., 2005)	45 cycles: 95 °C 30 s, 53 °C 45 s, 72 °C 45 s, 78 °C 20 s	35 cycles: 94 °C 30 s, 53 °C 45 s, 72 °C 10 min

### 2.5 Microbial community composition

Prokaryotic 16S rRNA and *amoA* genes were PCR-amplified as described in Rasche et al. (2011) (Table 1). Following the results of qPCR analysis (no treatment effects (“Foxy-2” and “organic inputs”) over the two seasons), we conducted T-RFLP analysis only in the second season based on the hypothesis that microbial community composition is less sensitive in responding to environmental changes than community abundance (Ritz et al., 2009, Wessén and Hallin, 2011). All forward primers were labeled with 6-carboxyflourescein at their 5′ ends. Replicate amplicons of the two genes were pooled, purified (Sephadex G-50, GE Healthcare Biosciences, Waukesha, WI, USA) according to Rasche et al. (2006b), and 200 ng of each purified amplicon were digested with a 5 U combination of enzymes *Alu*I and *Rsa*I (New England Biolabs, Ipswich, USA). Reactions were incubated at 37 °C for 4 h, and purified (Sephadex G-50). An aliquot of 2 μl was mixed with 17.75 μl HiDi formamide (Applied Biosystems) and 0.25 μl internal 500 ROX™ size standard (Applied Biosystems). Labeled terminal-restriction fragments (T-RFs) were denatured at 95 °C for 3 min, chilled on ice and detected on an ABI 3130 automatic DNA sequencer (Applied Biosystems). Peak Scanner™ software package (version 1.0, Applied Biosystems) was used to compare relative lengths of T-RFs with the internal size standard and to compile electropherograms into numeric data set, in which fragment length and peak height > 50 fluorescence units were used for profile comparison. T-RFLP profiles used for statistical analyses were normalized according to Dunbar et al. (2000).

### 2.6 Soil chemical analysis

Soil chemical analyses were performed on all soil samples taken at EC30 and EC90. Total carbon (TC), total nitrogen (N_t_), extractable organic C (EOC), extractable N (EON) and pH of soils were recorded on bulk soils. The pH analysis was conducted in a soil water ratio of 1:2.5 using a pH meter (inoLab^®^ Labor-pH-Meter, WTW GmbH, Weilheim, Germany). TC and N_t_ was quantified by dry combustion (vario MAX CN analyzer, Elementar Analysensysteme GmbH, Hanau, Germany). For EOC measurement, 5 g of soil were extracted with 20 ml 0.5 M K_2_SO_4_, shaken horizontally (250 rpm) for 30 min and filtered (Rotilabo-Rundfilter AP55.1 (retention of 2–3 μm), Carl Roth GmbH, Karlsruhe, Germany). EOC concentration in filtered extract was measured on an Analytik Jena Multi N/C 2100 analyzer (Analytik Jena AG, Jena, Germany). Ammonium (NH_4_^+^) and nitrate (NO_3_^−^) were extracted with 1 M KCl (soil to extractant ratio (w/v) of 1:4), shaken on a horizontal shaker for 30 min at 250 rpm and filtered (Rotilabo-Rundfilter AP55.1, Carl Roth GmbH). Concentrations of NH_4_^+^ and NO_3_^−^ were measured on an auto-analyzer (Bran & Luebbe, Norderstedt, Germany) (Mulvaney, 1996), while EON was determined as the difference between N_t_ and mineral N (NH_4_^+^ and NO_3_^−^) according to Rousk and Jones (2010).

### 2.7 Statistical analysis

Statistical analyses were performed using Statistical Analysis Software program (SAS Institute, 2015). The analysis was done using a generalized linear mixed model assuming a negative binomial error distribution and a log link function. The fixed effects were; “Treatment” (“Foxy-2”, organic N addition (*T. diversifolia)*, *S. hermonthica*, control), “Site” (soil properties), “Season” (SR 2012/2013, LR 2013), and “Growth stage” of maize (EC30, EC60, EC90) and all their two and three way interactions. The random effect included the blocking factor and replicates nested within blocks. The full model was fitted using restricted log pseudo-likelihood in the SAS GLIMMIX procedure (SAS Institute, 2015). We included a variance-covariance matrix to account for temporal auto-correlation in the residuals for all observations made within a block. The model was fitted separately for all variables across sites (soil properties) (Table 2). Due to the observed significant site effect the model was further fitted separately per site to account for site differences. Adjusted means and their associated standard errors at 95% confidence limits were estimated on the original data scale. Wherever, the two- or three-way interactions were significant, we decomposed them in terms of their simple effects slices. This enabled us to test adjusted means for significance between pairs of treatment, season or crop growth stages at fixed values of the other terms in the interaction effect. The means were compared using the PDIFF option of the LSMEANS as well as the letters (letter display) from the SAS generalized linear mixed models procedure. For soil chemical properties, NH_4_^+^ and EON were log base 10 transformed, while NO_3_**^−^** was square root transformed. Therefore, no standard errors are shown for these values as back transformed data was used for comparison purposes with the non-transformed chemical data (Table 4) (Piepho, 2009).

**Table 2:** Analysis of variance to determine significant effects of factors ‘Site’ (ST), “Season” (SS), “Treatment” (T) and “Growth stage” (EC) and their interactions on soil chemical properties and abundance of the two assayed genes.

Factor	Total bacteria	Total archaea	Bacterial *amoA* gene	Archaeal *amoA* gene	EOC (mg kg^−1^)	EON (mg kg^−1^)	NH_4_^+^ (mg kg^−1^)	NO_3_^+^ (mg kg^−1^)	pH (H_2_O)
Season (SS)	n.s.	***	***	***	***	***	***	***	***
Treatment (T)	n.s.	n.s.	n.s.	n.s.	n.s.	n.s.	n.s.	*	***
Site (ST)	n.s.	***	n.s.	n.s.	*	**	***	***	***
Growth stage (EC)	***	***	***	***	***	***	***	***	***
SS × T	n.s.	*	n.s.	n.s.	n.s.	*	n.s.	n.s.	***
SS × ST	***	n.s.	***	n.s.	n.s.	***	*	***	***
ST × T	n.s.	n.s.	n.s.	n.s.	n.s.	n.s.	**	n.s.	***
EC × T	n.s.	n.s.	***	n.s.	**	n.s.	**	*	***
EC × ST	***	***	***	**	***	*	***	**	***
SS × T × ST	*	n.s.	n.s.	n.s.	n.s.	n.s.	n.s.	*	***
EC × T × ST	n.s.	n.s.	***	n.s.	n.s.	n.s.	n.s.	**	***

Significance levels: n.s.: P > 0.05; *P < 0.05; **P < 0.01; ***P < 0.001.

Pearson’s linear correlation coefficients were calculated for assessing the relations between abundance of nitrifying and total prokaryotes with soil chemical properties.

“Treatment”, “Site”, and “Growth stage” effects as well as their interaction effects on T-RFLP data sets for each gene were tested using permutation multivariate analysis of variance (PERMANOVA) (Anderson and Walsh, 2013). Factor effects were further assayed based on Bray-Curtis similarity coefficients (Rees et al., 2005, Rasche et al., 2011, Rasche et al., 2014). A similarity matrix was generated for all possible pairs of samples for each target gene. The similarity matrix was used for analysis of similarity (ANOSIM) to test the hypothesis that composition of studied microbial communities was altered by factors “Treatment”, “Site”, and “Growth stage”. ANOSIM is based on rank similarities between the sample matrix and produces a test statistic ‘*R*’ (Rees et al., 2005). A ‘global’ *R* was first calculated in ANOSIM, which evaluated the overall effect of a factor in the data set. This step was followed by a pairwise comparison, whereby the magnitude of *R* indicated the degree of separation between two tested communities. An *R* score of 1 indicated a complete separation, while 0 indicated no separation (Rees et al., 2005). For graphical visualization of the distinct effect of factor “Growth stage” and “Site” on the composition of analyzed prokaryotic communities, canonical analysis of principal coordinates (CAP) was performed on resemblance matrix data generated based on Bray-Curtis similarity coefficients (Clarke, 1993, Anderson et al., 2008, Muema et al., 2015). Moreover, to test the influence of soil chemical properties on the community composition shifts of assayed genes, distance-based linear models (DISTLM) were used (Permanova+ software package in Primer v6) (Anderson et al., 2008). This procedure calculates a linear regression between the community composition and log transformed soil chemical data using the Shannon diversity index (H’) to evaluate how much of the variation in the microbial community composition is explained by variation in the soil chemical data (Legender and Andersson, 1999, Boj et al., 2011). PERMANOVA, ANOSIM, CAP and DISTLM analyses were conducted with Primer6 for windows (version 6.1.13) (Primer-E Ltd., Ply-mouth, UK) with PERMANOVA+ version 1.0.6 as add-on for Primer6 software (Anderson et al., 2008).

## 3 Results

### 3.1 Microbial abundance

**Fig. 2:**
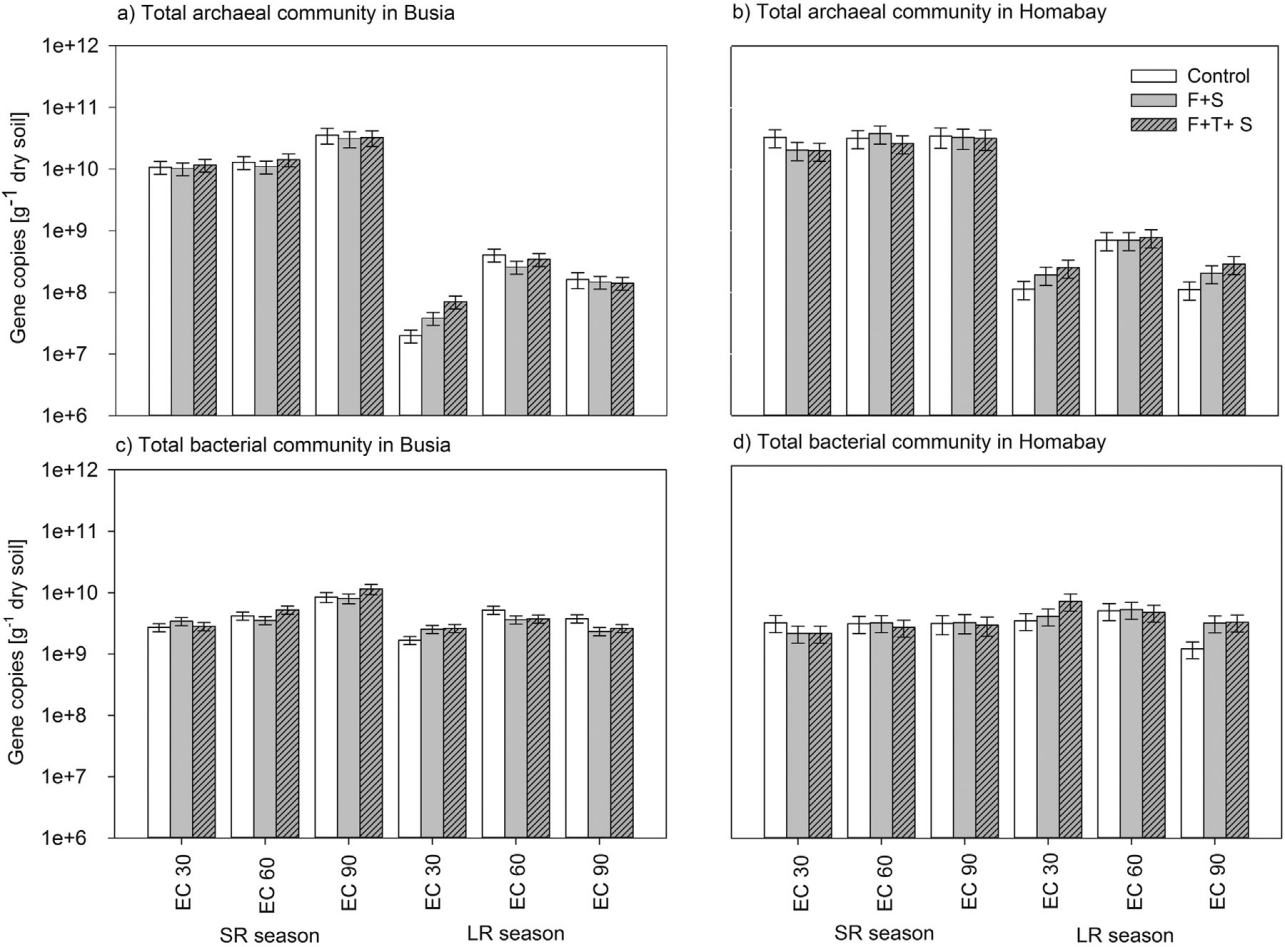
Abundance of the total archaeal and bacterial community at Busia (a, c) and Homabay (b, d) sites as determined during the two cropping seasons. Values are given as average (*n* = 3) along with standard error (SE). Treatments are: C + S, uncoated maize and *S. hermonthica*, F + S, coated maize with “Foxy-2” and *S. hermonthica*, F + S + T, coated “Foxy-2”, *S. hermonthica* and *T. diversofolia*.

Abundance of archaeal 16S RNA genes was influenced by factors “Site”, “Season” and “Growth stage” (Table 2 and Fig. 2a and b). Overall, Homabay site had higher 16S RNA archaeal gene copies than Busia site (P < 0.001, Fig. 2a and b). A clear distinction of archaeal abundance was revealed in response to the three crop growth stages, where EC90 showed generally highest 16S RNA archaeal gene copies during the SR season, while archaeal abundance was dominating during EC60 during the LR season (P > 0.001) (Fig. 2a and b). Furthermore, a higher archaeal 16S RNA gene abundance was found in Homabay at EC30 and EC60 in both seasons compared to Busia (P < 0.001) (Fig. 2a and b).

Total bacterial abundance was most significantly influenced by factor ‘Growth stage’ resulting in lowest bacterial 16S RNA gene copies at EC30 (Fig. 2c and d). Additionally, an interaction was found between factors “Season” and “Site”, where higher bacterial 16S rRNA gene copies were measured at Busia during SR season (P < 0.001) (Table 2 and Fig. 2c). The interaction of “Growth stage” and “Site” revealed a higher significant increase in bacterial 16S RNA gene copies from EC30 to EC60 and EC90 at Busia during SR season compared to LR and Homabay (P < 0.001) (Table 2 and Fig. 2c and d).

**Fig. 3:**
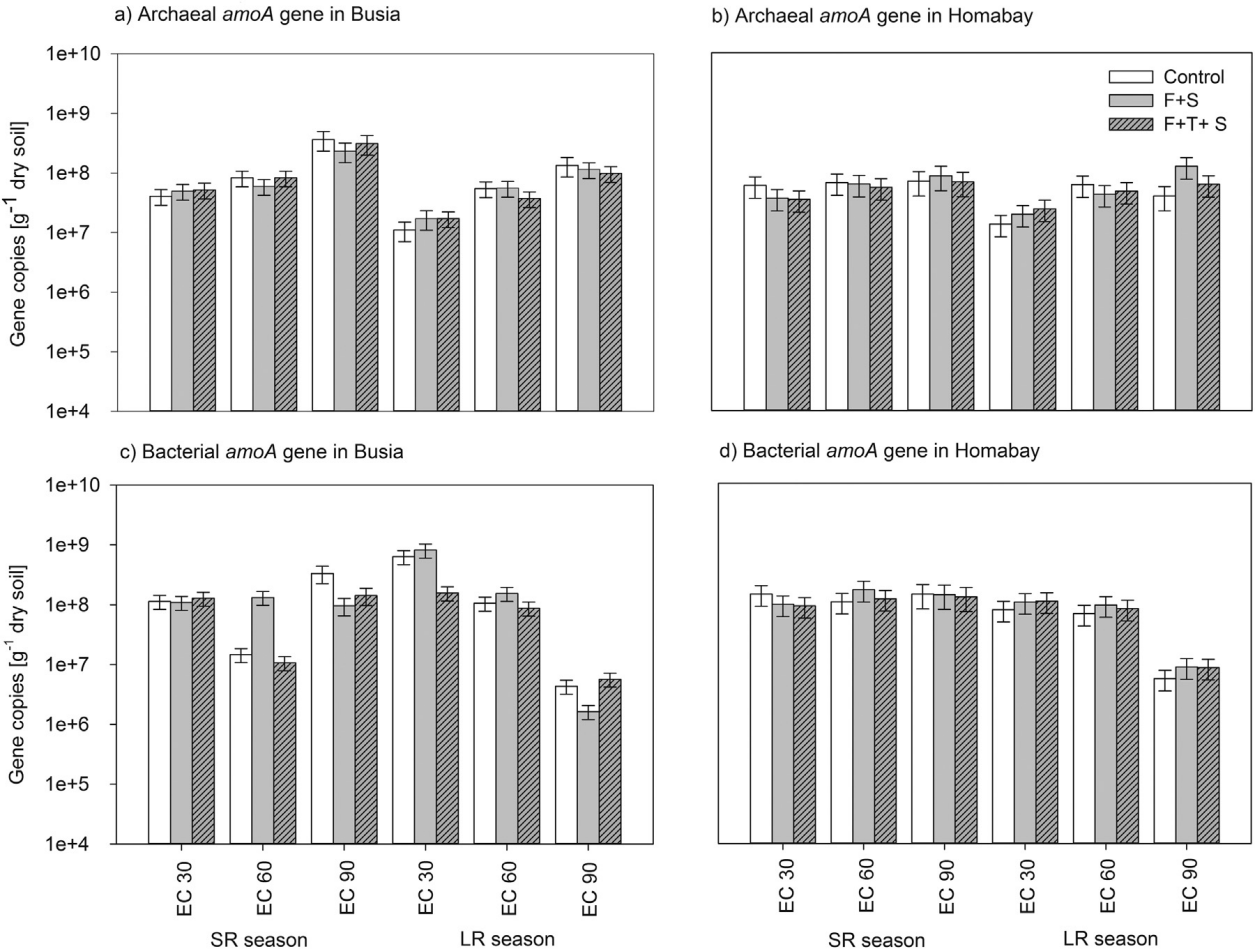
Abundance of the archaeal and bacterial *amoA* genes at Busia (a, c) and Homabay (b, d) sites as determined during during the two cropping seasons. Values are given as average (*n* = 3) along with standard error (SE). Treatments are: C + S, uncoated maize and *S. hermonthica*, F + S, coated maize with “Foxy-2” and *S. hermonthica*, F + S + T, coated “Foxy-2”, *S. hermonthica* and *T. diversofolia*.

Abundance of archaeal *amoA* gene abundance (AOA) was influenced by factors “Season” and “Growth stage” and significant interactions were found between “Growth stage” and “Site” (P < 0.05) (Table 2). SR season showed higher AOA abundance in comparison to LR season particularly at Busia, where also highest gene copies were found at EC90 in both seasons (P < 0.01) (Fig. 3a).

Abundance of bacterial *amoA* gene abundance (AOB) was influenced by factors “Season” and “Growth stage” revealing a significant interactions of “Growth stage” “Treament” and “Site” (P < 0.001) (Table 2). A major trend was that during the LR season, AOB revealed a constant abundance decrease from EC30 to EC90 and more strongly so at Busia (Fig. 3c). Generally, all genes tended to show a higher abundance in the treatments F + S (“Foxy-2”) and its combination with *Tithonia diversifolia* (F + S + T) than the control (C) at EC30 during the LR season in both sites (P > 0.05) (Figs. 2 a,b,c,d and 3 a,b,d).

### 3.2 Microbial community composition

**Fig. 4:**
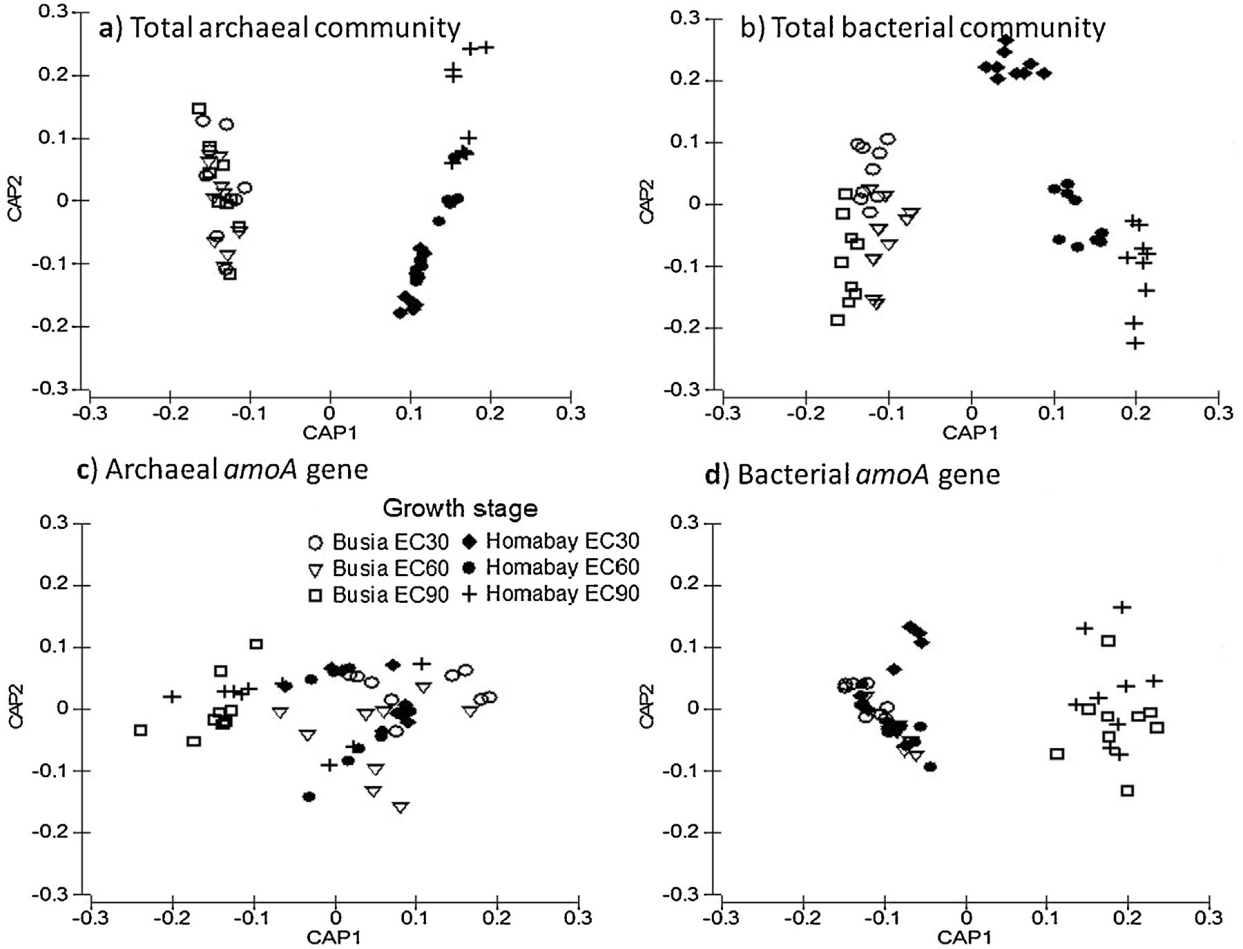
Canonical analysis of principal coordinates (CAP) for visual presentation of prokaryotic community composition (TRLFP) as shaped by “Site” and “Growth stage”. Prokayotic communities are (a) total archaea, (b) total bacteria, (c) ammonia-oxidizing archaea and (d) ammonia-oxidizing bacteria.

Analysis of similarity (ANOSIM) of T-RFLP data revealed significant effects of factors “Site” and “Growth stage” but not “Treatment” on the community composition of all studied genes (P < 0.05) (Table 3). A subsequent PERMANOVA analysis revealed significant interactions (P < 0.001) as evidenced by the effect of “Growth stage” on total archaeal communities at Homabay but not at Busia site which was further proved by pairwise ANOSIM (EC30 versus EC60 (R = 0.234), EC60 versus EC90 (R = 0.44), EC30 versus EC90 (R = 0.694) (P < 0.01)) (Fig. 4a). For total bacterial community, effect of “Growth stage” was more pronounced at Homabay (Fig. 4b) as was evidenced by pairwise ANOSIM (EC30 versus EC60 (R = 0.735), EC30 versus EC90 (R = 0.941) and EC60 versus EC90 R = 0.268). Likewise, the AOB (*amoA* gene) community composition revealed similar trends (Fig. 4d). Conversely, AOA (*amoA* gene) community composition differences by “Growth stage” were observed to a greater extent at Busia mainly between EC30 and EC90 (R = 0.9) (P < 0.001) (Fig. 4c).

**Table 3:** Global R values for the main factors “Site”, “Growth stage” and “Treatment” as obtained from the analysis of similarity of T-RFLP fingerprints generated from the four studied genes.

Prokaryotic group	Factor		
	Site	Growth stage	Treatment
Total bacteria	0.791^a^***	0.780***^b^	−0.038^n.s.^
Total archaea	0.950***	0.708***	−0.046^n.s.^
Bacterial *amoA* gene	0.172**	0.400***	−0.009^n.s.^
Archaeal *amoA* gene	0.648***	0.132**	−0.015^n.s.^

a R indicates the degree of separation between two populations, with a score of 1 indicating complete separation and 0 indicating no separation.

b Significance levels: n.s.: P > 0.05; *P < 0.05; **P < 0.01; ***P < 0.001.

### 3.3 Soil chemical properties

EOC and EON were influenced by factors “Season” “Growth stage” and “Site” with significant interactions of factor ‘Growth stage’ and “Site” (P < 0.05) (Table 2). EOC was highest at EC90 at Busia during SR season (P < 0.05) (Table 4), while EON was largest at Homabay site at EC30 of SR season (P < 0.05) (Table 4). EON values were highest at Busia at EC30 during LR season (Table 4). For mineral N values, seasonal effects were evidenced by higher NH_4_^+^ concentrations at EC30 during SR season (P < 0.001) (Table 4). No consistent effect of factors ‘Treatment’, “Site” and “growth stage” on NO_3_^−^ contents was observed due to several interactions (Table 2). Soil pH was influenced by all four factors with significant interactions (P < 0.001) (Table 2). For example, soil at Homabay had a higher pH compared to Busia, and SR season induced mostly higher soil pH at both sites (Table 4).

**Table 4:** Dynamics of soil chemical properties as driven by factors “Site”, “Season”, “Treatment” and “Growth stage” and their interactions. Values are given as average (*n* = 3) along with standard error (SE). Different letters within a column show significant differences within sites and growth stages EC30 and EC90 (P < 0.05). Values without SE have been back transformed.

Site	Growth stage	Treatment	EOC (mg kg^−1^)		EON (mg kg^−1^)		NH_4_^+^ (mg kg^−1^)		NO_3_^−^ (mg kg^−1^)		pH (H_2_O)	
			SR	LR	SR	LR	SR	LR	SR	LR	SR	LR
Busia	EC30	C + S	242a	273a	22ab	118a	71a	35b	27a	40b	5.48a	5.33c
		F + S	264a	247a	14b	203a	97a	81a	29a	101a	5.49a	5.41b
		F + S + T	294a	264a	31a	132a	67a	52ab	36a	55b	5.35b	5.43a
		SE	12.54	12.43							0.02	0.02

	EC90	C + S	281a	197b	37a	48.7a	13a	13a	0.27a	36a	5.64c	4.91b
		F + S	283a	256a	34a	92.3a	13a	21a	1.07a	15b	5.70b	4.86c
		F + S + T	301a	224ab	26a	69.9a	14a	16a	0.61a	20b	5.81a	4.99a
		SE	9.73	13.46							0.03	0.02

Homa Bay	EC30	C + S	294a	295a	204a	148a	123a	76a	34a	18a	7.04b	6.68a
		F + S	263a	226b	142ab	150a	143a	89a	38a	14a	6.91c	6.17b
		F + S + T	266a	281ab	92b	198a	99a	82a	16b	14a	7.26a	6.11c
		SE	10.04	14.13							0.05	0.09

	EC90	C + S	273a	156a	48a	25a	4b	3ab	9a	5a	7.35b	7.59a
		F + S	221ab	172a	33a	22a	4b	2b	5a	5a	7.93a	7.22c
		F + S + T	214b	178a	40a	29a	13a	4a	2a	5a	7.27c	7.41b
		SE	15.45	4.29							0.10	0.05

Treatments: C + S = uncoated maize + *S. hermonthica*, F + S = coated maize (with “Foxy-2”) + *S. hermonthica*, F + S + T = coated maize + *S. hermonthica* + *Tithonia diversofolia*; Season: SR = short rains, LR = Long rains; Growth stage: EC30 = Early leaf development stage, EC90 = Senescence stage; SE: Standard error.

### 3.4 Correlations between microbial abundance and soil chemical properties

Extractable organic carbon (EOC) was positively correlated with total bacterial abundance and AOB abundance (P < 0.05), whereas extractable organic nitrogen (EON) negatively correlated with AOA (P < 0.001) (Table 5). Mineral N (ammonium (NH_4_^+^) and nitrate (NO_3_^−^)) revealed a negative correlation with AOA abundance (P < 0.01). In addition, NO_3_^−^ correlated negatively with total bacterial abundance (P < 0.05). Soil pH positively correlated with total archaeal abundance (P < 0.001).

**Table 5:** Pearson’s linear correlation coefficients between abundance of the two genes and soil chemical data obtained in Homabay and Busia sites at EC30 and EC90.

Prokaryotic group	EOC (mg kg^−1^)	EON (mg kg^−1^)	NH_4_^+^ (mg kg^−1^)	NO_3_^+^ (mg kg^−1^)	pH (H_2_O)
Total bacteria	0.253*	n.s.	n.s.	−0.325*	n.s.
Total archaea	n.s.	n.s.	n.s.	n.s.	0.365**
Bacterial *amoA* gene	0.263*	n.s.	n.s.	0.320*	n.s.
Archaeal *amoA* gene	n.s.	−0.387**	−0.418**	−0.374*	n.s.

Significance levels: n.s.: P > 0.05; *P < 0.05; **P < 0.01; ***P < 0.001.

### 3.5 Regressions between microbial community composition and soil chemical properties

Variance in total bacterial community composition was explained by alterations of extractable organic carbon (EOC), extractable organic nitrogen (EON) and ammonium (NH_4_^+^) concentrations (Table 6). Changes in total archaeal community composition were explained by alterations of all measured soil chemical properties, with a greater variation explained by nitrate (NO_3_^−^) (Table 6). AOB community composition changes were explained all measured soil chemical properties, except pH (Table 6). However, a greater percentage of AOB was explained by NH_4_^+^ (46%) (Table 6). Community composition changes of AOA were mainly explained by soil pH (Table 6).

**Table 6:** Regression analyses between community composition of the two studied genes and soil chemical data taken at EC30 and EC90 across both study sites.

Prokaryotic group	EOC (mg kg^−1^)	EON (mg kg^−1^)	NH_4_^+^ (mg kg^−1^)	NO_3_^+^ (mg kg^−1^)	pH (H_2_O)
Total bacteria	0.280^a^***^b^	0.186^a^**^b^	0.383***	0.026n.s.	0.000n.s.
Total archaea	0.107*	0.107*	0.231***	0.316***	0.168**
Bacterial *amoA* gene	0.344***	0.245**	0.459***	0.273**	0.001n.s.
Archaeal *amoA* gene	0.000n.s	0.009n.s.	0.010n.s.	0.0715n.s.	0.295**

a R^2^ indicates the proportion of variation in Shannon diversity explained by log values of the chemical data.

b Significance levels: n.s.: P > 0.05; *P < 0.05; **P < 0.01; ***P < 0.001.

## 4 Discussion

### 4.1 Dominance of environmental factors over presence of BCA

Previous studies on non-target effects of beneficial *Fusarium* spp. strains as biocontrol agents (BCAs) on soil microbial communities have been restricted to controlled conditions with short investigation periods rather than field conditions (Edel-Hermann et al., 2009, Karpouzas et al., 2011, Musyoki et al., 2015). Generally, controlled conditions do not represent the natural conditions that prevail in the rhizopshere of crops grown in the field. In this respect, the tripartite interaction between *Fusarium* spp. BCAs, indigenous microbial communities and host crop (rhizosphere) have been suggested to vary with environmental conditions (Goh et al., 2009, Mendes et al., 2013). To account for this, we carried out field experiments during two cropping seasons to study the effects of the BCA “Foxy-2” relative to the acknowledged effects of natural factors including soil properties (site), crop growth stage and seasonality (rainfall patterns) on the abundance (qPCR) and community composition (T-RFLP) of total and nitrifying prokaryotes in rhizospheres of maize grown in contrasting soils. Our results revealed that site, crop growth stage and seasonal variations controlled the abundance and community composition of prokaryotic nitrifiers to a greater extent than “Foxy-2” inoculation. This central outcome further reinforced the need for extended field studies under different conditions for better understanding of rhizospheric interaction effects between indigenous communities and potential biological control agents (Musyoki et al., 2015).

In the current study, Busia and Homabay soils had clay contents ranging from 33 to 49%, respectively, which were higher than that of the sandy soil (22%) used for a previous study by Musyoki et al. (2015), where a promoting effect nitrifying archaea was reported in a sandy but not in a clay soil. These soil texture differences may have partially masked “Foxy-2” related shifts in the abundance of total and also nitrifying prokaryotes. Notably, total archaeal abundance was higher in the Homabay soil than in that of Busia, while total bacteria, nitrifying archaea (AOA) and bacteria (AOB) were not influenced by soil type (site). This higher abundance and also a clear distinction of the composition of the total archaeal community in the clayey Homabay soil was mainly attributed to the higher soil pH and higher clay contents promoting a larger soil organic carbon (SOC) background at Homabay, an explanation which is in agreement with earlier studies (Gerzabek et al., 2002, Shen et al., 2008, Morimoto et al., 2011). Likewise, we have recently observed that clayey soils hampered the proliferation of “Foxy-2” (Zimmermann et al., 2015). It is likely that the archaeal community colonized similar resource niches like “Foxy-2” which has been reported to proliferate better in low nutrient conditions of e.g. sandy soils (Larkin and Fravel, 2002, Erguder et al., 2009, Zimmermann et al., 2015). We therefore speculated that indigenous rhizosphere archaea were involved in the suppression of “Foxy-2” as was not only corroborated by negative correlations between the BCA and indigenous total archaea (J. Zimmermann, personal communication), but also by earlier studies stating that clayey soils evolve a high natural suppression potential against microorganisms (Toyota et al., 1996, Larkin and Fravel, 2002). This hypothesized suppressive effect by archaea became obvious during the later stages of the vegetation period in both seasons (i.e., SR (short rains), LR (long rains)), where abundance of total archaea but also that of AOA increased significantly. We assumed that this archaeal dominance at the later crop growth stages increased their competitive and hence suppressive abilities over “Foxy-2”. This niche-based resource competition may have been particularly evident at EC90, when “Foxy-2” was in its saprophytic stage and, similar to indigenous rhizosphere archaea, further increasing the capitalization on available indigenous SOC as central resource (Kandeler et al., 2002, Garbeva et al., 2004, Elzein et al., 2010, Musyoki et al., 2015). Moreover, advanced decomposition of organic matter derived from the fallow period prior to the field experiment setup in the SR season may have contributed to this abundance boost of AOA over AOB since AOA are generally more adapted to acquisition of organic derived nutrients over their bacterial counterparts (Wessén et al., 2010).

### 4.2 Foxy-2 did not induce a resource limitation for bacteria and archaea

Clear indications of resource limitation for bacterial and archaeal abundances were found under the tested field conditions. This was most obvious at EC30 during LR, where abundance of total and nitrifying bacteria and archaea increased with application of *Tithonia diversifolia* (TD) residues over the control treatment (C). However, this apparent resource limitation was not induced by presence of “Foxy-2” (F) as there was firstly a positive effect of the BCA on nitrifying (*amoA*) and total bacterial (16S) as well as their archaeal abundances, and the positive effect was even greater when combined with TD. This stimulating effect was not observed during the initial SR which was traced back to the decomposition of organic matter derived from the fallow period prior to the field experiment setup in the SR season. In addition, the high rainfall amount during the SR rain season (149 mm month^−1^) in comparison to the lower rainfall amount during the LR season (109 mm month^−1^) may have contributed to indirect changes through rhizodeposition, consequently affecting the organic resource availability in the rhizosphere (Bell et al., 2009, Rasche et al., 2011). This resource excess was reflected by overall higher total bacterial and archaeal abundances during the SR season.

Furthermore, AOB abundance dominated at the earlier growth stages, particularly at EC30 which was linked to the provision of easily degradable rhizodeposits including EOC, as well as nitrogenous metabolites such as extractable organic nitrogen (EON) and nitrates (NO_3_^+^) correlating positively with AOB, but negatively with AOA abundance (Hai et al., 2009, Aira et al., 2010, Neal et al., 2012). Similarly, a strong community differentiation was found between AOB and AOA at EC30 and EC90 corroborating the colonization of distinct ecological niches. In this respect, we found that ammonium (NH_4_^+^) contents explained more than 40% of the AOB community shift, while soil pH was the only parameter explaining the AOA community shift. Our findings agreed with earlier studies stating that niche differentiation between AOA and AOB is mainly driven by alterations of soil N and pH conditions (Valentine, 2007, Shen et al., 2008, Erguder et al., 2009, Wessén et al., 2010), which were not consistently significantly influenced by presence of “Foxy-2”.

## 5 Conclusion and outlook

Our field study revealed that “Foxy-2” application did not impose a negative effect on the abundance and community composition of total and nitrifying prokaryotes. This is an important prerequisite concerning the registration of “Foxy-2” as a potential *S. hermonthica* BCA. The major observation that crop growth stage, seasonal variations and soil properties controlled total and nitrifying prokaryote abundance and community composition to a greater extent than “Foxy-2” inoculation demonstrates the need to include field studies over several seasons to obtain a more detailed, site-and seasonality-specific understanding on non-target effects of potential BCAs on indigenous microbial communities colonizing the rhizosphere of *S. hermonthica* affected crops.

Notably, we found clear indications that particularly archaea and “Foxy-2” colonized similar ecological niches for organic resource acquisition in the rhizospheres of maize grown in clayey soils. In that respect, we postulate that archaeal domination in clayey soils is a considerable regulator of “Foxy-2” proliferation due to their greater rhizosphere competence over that of “Foxy-2”. It needs, however, to be tested if this potential out competition of archaea holds true under other environmental conditions than those tested in this study. Therefore, progressive studies should emphasize surveys in distinct agro-ecological zones in which not only the dynamics of indigenous rhizosphere communities under the presence of “Foxy-2” will be assayed. These studies should also consider local adaptation mechanisms of “Foxy-2” which might induce a higher resource competition potential against natural prokaryotic and also fungal communities such as sandy nutrient limited soils. Under such conditions, the use of N-rich organic residue inputs may be considered to compensate any resource competition.
